# Abcès pottique intra médullaire: à propos d’un cas

**DOI:** 10.11604/pamj.2019.34.186.20838

**Published:** 2019-12-06

**Authors:** Haboubacar Chaibou Sode, Assoumane Ibrahim, Hounkpatin Seton Stachys Beranger, Nikiema Habi Alizéta Soong-Meenga, Kelani Aminath, Sanoussi Samuila

**Affiliations:** 1 Service de Neurochirurgie, Hôpital National de Niamey, Niamey, République du Niger; 2 Service de Neurochirurgie, Hôpital de Référence de Maradi, Maradi, République du Niger; 3 Faculté des Sciences de la Santé, Université d’Abdou Moumouni de Niamey, Niamey, République du Niger

**Keywords:** Pott multifocal, abcès intra médullaire, chirurgie, Multifocal Pott's disease, intramedullary abscess, surgery

## Abstract

La tuberculose du système nerveux central est une affection rare. Elle se présente sous plusieurs formes mais l'association d'un abcès intra médullaire et un pott multifocal est exceptionnelle. Nous rapportons le cas d'une patiente âgée de 23 ans chez qui le diagnostic d'un pott multifocal L2-L3 et L4-L5 a été posé sur la tomodensitométrie (TDM) du rachis. Elle a été mise sous traitement anti tuberculeux et devant l'installation d'un tableau de compression médullaire deux mois après le début du traitement, une imagerie par résonance magnétique (IRM) médullaire a été réalisée objectivant une image d'abcès intra médullaire à hauteur de T4. Elle a été opérée bénéficiant dans le même temps opératoire du drainage de l'abcès intra médullaire et de la stabilisation du rachis. L'évolution a été favorable, la patiente a été suivie pendant 12 mois. L'association d'un abcès intra médullaire et un pott multifocal est exceptionnelle. Le traitement repose sur les antituberculeux et la chirurgie pour lever la compression médullaire et ou stabiliser le rachis le cas échéant.

## Abstract (en)

Central Nervous System (CNS) Tuberculosis (TB) is rare. It manifests in many forms, but the association between intramedullary abscess and multifocal Pott's disease is exceptional. We report the case of a 23-year old female patient diagnosed with multifocal Pott's disease at L2-L3 and L4-L5 based on computed tomography (CT) scan of the spine. She received antituberculous treatment. Two months after the beginning of treatment spinal cord compression occurred. Medullary magnetic resonance imaging (MRI) was performed, which showed intramedullary abscess at T4. She underwent surgery with drainage of intramedullary abscess and spinal stabilization. Outcome was favorable, during the 12-month follow-up. The association between intramedullary abscess and multifocal Pott's disease is exceptional. Treatment is based on anti-tuberculous drugs, surgery for spinal decompression and/or spine stabilization if possible.

## Introduction

La tuberculose ostéo articulaire est rare représentant seulement 1 à 3% de tous les cas de tuberculose [1]. La tuberculose rachidienne se présente sous plusieurs formes: le mal de Pott ou spondylodiscite tuberculeuse qui est la plus fréquente (60%) suivie de l'arachnoidite (20%), de la méningite (12%), et des lésions intra médullaires (8%) [2]. Les abcès intra-médullaires sont extrêmement rares [3]. L'association d'un abcès intra médullaire et un pott multifocal est exceptionnelle, à cette date aucun cas n'a été rapporté dans la littérature à notre connaissance. Les signes cliniques peuvent être de la douleur aux signes de compression médullaire. Le bilan paraclinique repose sur la TDM et l'IRM médullaire et la confirmation tuberculeuse sur l'examen anatomopathologique. Le traitement repose sur les antituberculeux et la chirurgie pour lever la compression médullaire et stabiliser le rachis le cas échéant. Nous rapportons le cas d'une patiente présentant un abcès intra médullaire associé à une double localisation d'une spondylodiscite traitée par la chirurgie et les antituberculeux.

## Patient et observation

**Figure 1:**
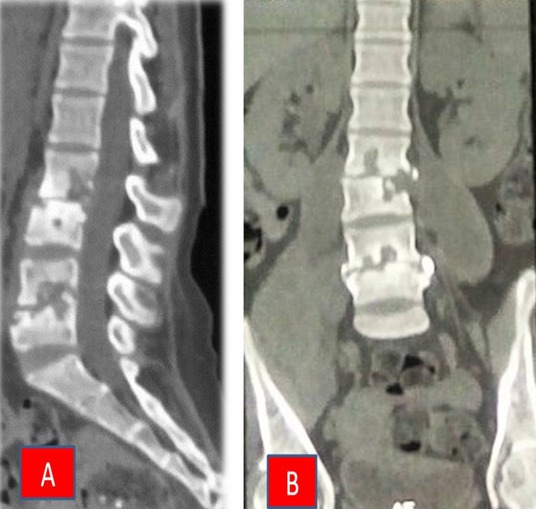
TDM lombaire: (A) reconstruction sagittale; (B) reconstruction coronale qui on objective la destruction disco corporeale L2-L3 et L4-L5

Il s'agit d'une patiente âgée de 23 ans sans antécédents pathologiques particuliers qui avait présenté des douleurs lombaires remontant à 6 mois pour lesquels elle a fait plusieurs séances de kinésithérapie avec rémission. Après quelques semaines se sont installées progressivement, une faiblesse des membres inférieurs, une fièvre vespérale et une perte de poids. Elle consulte dans un service de médecine générale ou une tomodensitométrie lombaire a été réalisée objectivant une spondylodiscite lombaire bifocale L2-L3 et L4-L5 (Figure 1) pour laquelle un traitement antituberculeux a été mis en route selon le Protocole National de Lutte Contre la Tuberculose extra pulmonaire. Après deux mois de traitement anti tuberculeux (TBC) bien conduit la fièvre persistait et une paraplégie s'installe ce qui a motivé la patiente à consulter en neurochirurgie. A l'admission, l'examen clinique retrouve une patiente consciente, présentant un état général passable avec une asthénie, une anorexie et un léger amaigrissement; elle était apyrétique. L'examen de la colonne vertébrale retrouve une gibbosité lombaire à hauteur de L2.

**Figure 2:**
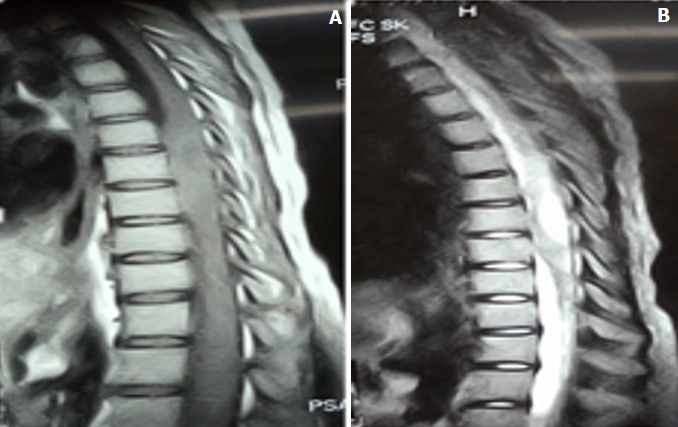
IRM pré opératoire en T1et T2 montrant abcès intra médullaire

On note un syndrome pyramidal fait d'une paraplégie flasque, une anesthésie en dessous de T4, une amyotrophie des membres pelviens, les réflexes ostéo tendineux au niveau des membres pelviens étaient vifs et un signe de Babinski bilatéral; avec quelques contractions involontaires de deux membres pelviens. Le bilan biologique objective une vitesse de sédimentation à 117, une anémie à 9g/dl. La radiographie pulmonaire était normale; une IRM médullaire a été réalisée objectivant un abcès intra médullaire se présentant en une lésion hypo-intense périphérique intra-médullaire (Figure 2) en séquence T1 et une hyper intensité centrale et un œdème péri lésionnel au niveau T4-T6 (Figure 2) en séquence T2. Pas de rehaussement après injection de gadolinium. Nous avons opéré la patiente, sous anesthésie générale, en décubitus ventral.

**Figure 3:**
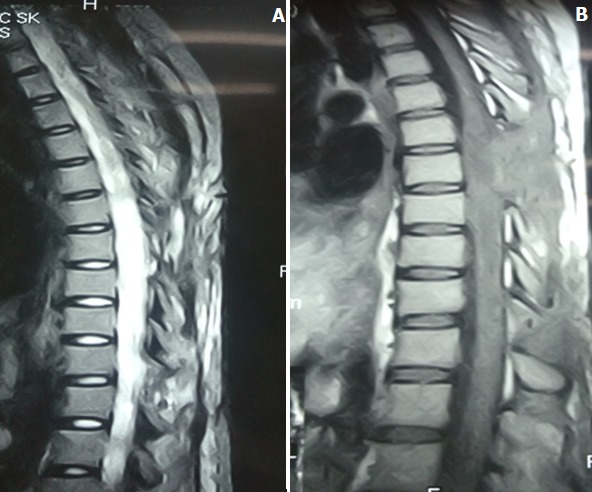
Images post opératoire: IRM en T2, T1

Dans un premier temps, nous avons réalisé une incision médiane dorsale en regard de T3 à T7 suivie d'une laminectomie étagée de T4 à T6. Après durotomie médiane, la moelle est apparue pleine et œdématiée ce qui nous a vite permis de reconnaitre la lésion qui était brunâtre. A l'aide d'un trocard nous avons ponctionné la lésion, ramenant du pus épais, de couleur brunâtre et un aspect caséeux. Nous avons procédé à l'ouverture et excision de la coque contenant le pus avant de refermer la dure mère. Dans un second temps nous avons réalisé une incision lombaire médiane en regard de L2 à L5 suivie d'une hémi laminectomie droite L2-L3 et L4-L5 L'ouverture de l'espace épidural a fait sourdre du pus épais, de couleur brunâtre et d'aspect caséeux; après l'évacuation du pus, suivi de l'excision et curetage de tissus nécrotique, nous avons rembourré la cavité de morceaux d'os provenant de hemi-laminectomie puis fermeture des différents plans. Les suites opératoires immédiates ont été simples, la patiente est sortie de l'hôpital après l'ablation des fils de suture au douzième jour. La patiente est sortie avec son traitement anti tuberculeux pour six mois et la prescription de séances de rééducation fonctionnelle. Le suivi post opératoire se faisait par rendez-vous mensuel à la consultation externe. L'IRM de contrôle avant la sortie confirme l'évacuation de l'abcès (Figure 3). La patiente a complètement récupéré à 12 mois post opératoire.

## Discussion

La spondylodiscite tuberculeuse ou mal de Pott, est l'atteinte de la colonne vertébrale incluant le sacrum et caractérisé par l'atteinte du disque intervertébral et des deux vertèbres adjacentes dans sa forme classique [4]; c'est la forme la plus fréquente des tuberculoses ostéoarticulaires [5] et représente la moitié des cas de tuberculoses osseuse [4, 6]. Elle s'étend très souvent dans les parties molles pré et latéro-vertébrales sous la forme d'abcès froid qui peut comprimer les organes de voisinage ou se fistuliser. Les abcès intra-médullaires sont extrêmement rares [3, 7], les quelques cas qui sont rapportés dans la littérature sont des cas cliniques. Communément il se produit par propagation hématogène de foyers d'infection lointains [8], Le tuberculome intramédullaire peut survenir après une dissémination hématogène, infection du liquide cérébrospinal (LCS), et très rarement par dissémination locale d'une tuberculose médullaire. L'atteinte médullaire de la tuberculose est le plus souvent secondairement à une localisation vertébrale; c'est la classique spondylodiscite à Bacille de Koch (BK) ou mal de Pott [9]. Sur le plan clinique il ressort qu'elle a présenté deux tableaux distinct, dans un premier temps une lombalgie clinique isolée et dont le bilan paraclinique orientait vers le pott multifocal L2-L3; L4-L5 puis deux mois plus tard l'installation progressive d'un tableau de compression médullaire nous a orienté vers une atteinte dorsal d'où la réalisation d'une IRM médullaire qui révèle l'abcès intra médullaire.

L'abcès intra médullaire est extrêmement rare comme l'atteste la littérature, il s'agit du premier cas dans notre contrée malgré la fréquence élevé de la maladie pottique et infectieuse en générale. Le diagnostic de mal de pott a été posé sur des arguments cliniques qui sont fièvre vespérale, altération de l'état général, cyphose rachidienne; sur le plan paraclinique, le scanner lombaire a objectivé une spondylodiscite étagée. Malgré la lésion évidente pottique lombaire, le syndrome de compression médullaire nous a vite permis de nous orienter vers une autre cause. La topographie dorsale de l'abcès est la plus retrouvée H Ennouali dans un case report retrouve l'abcès en T6-T7 également responsable d'un para parésie [10]. Sur le plan para clinique, l'avènement récent de l'IRM dans notre contrée a sans doute aider à faire ce diagnostic. La plus part des auteurs ont fait recours à cet examen.

Sur le plan thérapeutique, notre patiente a été pris en charge en un temps opératoire l'abcès intramédullaire et les lésions pottique ceci est du faite de l'habitude du service contrairement à certains auteurs qui préconisent l'abord antérieur [10, 11], Abderrazzak El Saqui [12] lui aborde l'abcès péri dural par voie postérieur mais réalise une laminectomie, nous nous faisons un abord postérieur qui consiste à une hémi laminectomie permettant l'évacuation de abcès épidural, résection des tissus contaminés sans déstabiliser d'avantage le rachis. Varatharajah S *et al.* [13] dans un article de revue de littérature ont rapporté que pour la localisation thoraco lombaire, ils commencent par une approche postérieure en stabilisant le rachis, une décompression par aspiration per cutanée du caséum diminue le risque de complications neurologiques post opératoires sur les patients à haut risque. Une approche antérieure par minimal invasive est utilisée pour réaliser un débridement optimal et une greffe osseuse afin de conserver la stabilité du rachis sur le long terme. Nous avons été renforcé de voir que macroscopiquement le pus pottique et intra médullaire sont de la même couleur. La disparition des signes d'imprégnation infectieuse avant l'intervention chirurgical qui intervient deux mois après le début de la thérapie antituberculeuse ne nous a pas poussé a insisté sur la bactériologie et l'examen anatomopathologique, Mazhar [13] a isolé un germe banal; *Escherichia coli* de l'abcès intra médullaire chez son patient qui est déjà sous thérapie antituberculeuse et a ajouter un antibiotique usuel. Nous avons continué les antituberculeux et la rééducation a permis d'accélérer la récupération neurologique.

## Conclusion

Le mal de Pott ou spondylodiscite tuberculeuse est la forme la plus fréquente de la tuberculose rachidienne mais de diagnostic souvent retardé. L'IRM médullaire reste un examen de tout premier ordre dans l'exploration des lésions intra médullaires tandis que la TDM est très utile dans l'évaluation des lésions osseuses vertébrales. Le pronostic est lié à la précocité diagnostic et une prise en charge médicale par l'anti TBC correcte accompagnée de la chirurgie en cas de compression médullaire et ou des lésions osseuses potentiellement instables.

## Conflits d’intérêts

Les auteurs ne déclarent aucun conflit d'intérêts.
